# 2-Meth­oxy­naphthalene-1,4-dione

**DOI:** 10.1107/S1600536811009883

**Published:** 2011-03-23

**Authors:** Bo Jin, Zhong-Cheng Song, Fu-Sheng Jiang, Wen-Hong Liu, Zhi-Shan Ding

**Affiliations:** a Department of Life Science, Zhejiang Traditional Chinese Medicine University, Hangzhou 310053, People’s Republic of China; b Bioengineering Department, Zhejiang Traditional Chinese Medicine University, Hangzhou 310053, People’s Republic of China

## Abstract

The title compound, C_11_H_8_O_3_, was isolated from *Impatiens balsamina* plants (balsam, LIB) grown in our laboratory. The two six-membered rings of the naphthalene-1,4-dione unit are coplanar [maximum deviation = 0.009 (1) Å]. The O and C atoms of the meth­oxy substituent also lie close to the naphthalene plane, with deviations of 0.0090 (2) and 0.047 (2) Å, respectively.

## Related literature

For background to compounds extracted from *Impatiens balsamina*, see: Ding *et al.* (2008 ▶). For the anti­microbial activity of flavonol and naphtho­quinone derivatives, see: Yang *et al.* (2001 ▶). For their anti-anaphylaxis properties, see: Yoshimi *et al.* (2003 ▶); Ishiguro *et al.* (1994 ▶) and for their use as anti-inflammatories, see: Hisae & Kyoko (2002 ▶). For standard bond-length data, see: Allen *et al.* (1987 ▶).
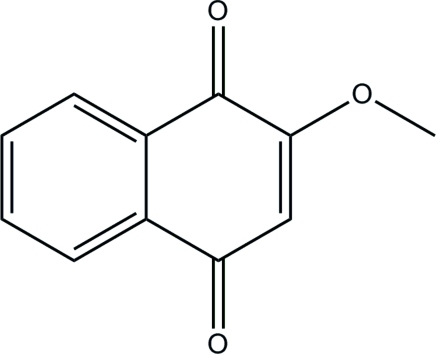


## Experimental

### 

#### Crystal data

C_11_H_8_O_3_
*M* *_r_* = 188.17
Monoclinic, 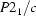

*a* = 3.904 (3) Å
*b* = 7.662 (6) Å
*c* = 28.81 (2) Å
β = 93.562 (7)°
*V* = 860.1 (12) Å^3^
*Z* = 4
Mo *K*α radiation
μ = 0.11 mm^−1^
*T* = 296 K
0.20 × 0.20 × 0.10 mm

#### Data collection

Enraf–Nonius CAD-4 diffractometer
Absorption correction: ψ scan (North *et al.*, 1968 ▶. *T*_min_ = 0.979, *T*_max_ = 0.989
7068 measured reflections
2082 independent reflections
1458 reflections with *I* > 2σ(*I*)
*R*_int_ = 0.032
3 standard reflections every 200 reflections  intensity decay: 1%

#### Refinement

*R*[*F* ^2^ > 2σ(*F* ^2^)] = 0.056
*wR*(*F* ^2^) = 0.136
*S* = 1.08
2082 reflections
128 parameters
H-atom parameters constrained
Δρ_max_ = 0.19 e Å^−3^
Δρ_min_ = −0.25 e Å^−3^

### 

Data collection: *CAD-4 Software* (Enraf–Nonius, 1989 ▶); cell refinement: *CAD-4 Software*; data reduction: *XCAD4* (Harms & Wocadlo, 1995 ▶); program(s) used to solve structure: *SHELXS97* (Sheldrick, 2008 ▶); program(s) used to refine structure: *SHELXL97* (Sheldrick, 2008 ▶); molecular graphics: *SHELXTL* (Sheldrick, 2008 ▶); software used to prepare material for publication: *SHELXL97*.

## Supplementary Material

Crystal structure: contains datablocks global, I. DOI: 10.1107/S1600536811009883/sj5115sup1.cif

Structure factors: contains datablocks I. DOI: 10.1107/S1600536811009883/sj5115Isup2.hkl

Additional supplementary materials:  crystallographic information; 3D view; checkCIF report

## Figures and Tables

**Fig. 1.**
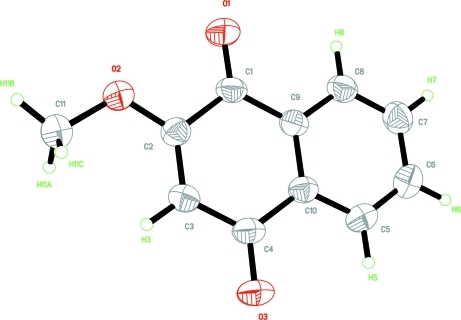
The molecular structure of the title compounds with atom labels and 50% probability displacement ellipsoids for non-hydrogen atoms.

## supplementary crystallographic information

### Comment

Modern chemical and pharmacological studies have identified flavonol and naphthoquinone derivatives, some of which have strong antimicrobial (Yang *et al.*, 2001) anti-anaphylaxis (Yoshimi *et al.*, 2003, Ishiguro *et al.*, 1994) and anti-inflammatory properties (Hisae & Kyoko, 2002). We have purified and identified an active component of the *Impatiens balsamina* plant (balsam, LIB) which was grown in our laboratory (Ding *et al.*, 2008) and authenticated by Professor Yao Zhensheng (Zhejiang Traditional Chinese Medicinal University). The molecular structure of the title compound is shown in Fig. 1. In the crystal, all bond lengths are within normal ranges (Allen *et al.*, 1987). The C1···C4,C9,C10 and C5···C10 rings of the naphthalene-1,4-dione unit are co-planar, maximum deviation 0.009 (1) Å. The O2 and C11 atoms of the methoxy substituent also lie close to the naphthalene plane with deviations of 0.0090 (2) Å and 0.047 (2) Å respectively.

### Experimental

Dried leaves (200 g)  of *Impatiens balsamina* were crushed, soaked with 55% alcohol (1500 ml) for 24 h and then reflux extracted for 40 min (1500 ml\3). Extracts were filtered and vacuum evaporated. In addition, 200 g of dried leaves were directly reflux extracted using chloroform (3000 ml\2). Next these extracts were filtered, combined, vacuum evaporated and the residue dried for further use. A portion of residue was re-chromatographed on silica gel using a petroleum ether-acetone (8:2) system and the isolated product was recrystallized from chloroform to yield the active component as light yellow crystals.

### Refinement

H atoms were positioned geometrically and refined using the riding-model approximation, with C—H = 0.93–0.97 Å, O—H = 0.82 Å, and *U*_iso_(H) = 1.2*U*_eq_(C) or *U*_iso_(H) = 1.5*U*_eq_(O).

### Crystal data

**Table:**

C_11_H_8_O_3_	*F*(000) = 392
*M**_r_* = 188.17	*D*_x_ = 1.453 Mg m^−^^3^
Monoclinic, *P*2_1_/*c*	Mo *K*α radiation, λ = 0.71073 Å
Hall symbol: -p 2ybc	Cell parameters from 1421 reflections
*a* = 3.904 (3) Å	θ = 3.0–26.7°
*b* = 7.662 (6) Å	µ = 0.11 mm^−^^1^
*c* = 28.81 (2) Å	*T* = 296 K
β = 93.562 (7)°	Block, yellow
*V* = 860.1 (12) Å^3^	0.20 × 0.20 × 0.10 mm
*Z* = 4	

### Data collection

**Table:**

Enrfa–Nonius CAD-4 diffractometer	1458 reflections with *I* > 2σ(*I*)
Radiation source: fine-focus sealed tube	*R*_int_ = 0.032
graphite	θ_max_ = 28.3°, θ_min_ = 2.8°
ω/2θ scans	*h* = −5→5
Absorption correction: ψ scan (North *et al.*, 1968.	*k* = −10→10
*T*_min_ = 0.979, *T*_max_ = 0.989	*l* = −38→36
7068 measured reflections	3 standard reflections every 200 reflections
2082 independent reflections	intensity decay: 1%

### Refinement

**Table:**

Refinement on *F*^2^	Primary atom site location: structure-invariant direct methods
Least-squares matrix: full	Secondary atom site location: difference Fourier map
*R*[*F*^2^ > 2σ(*F*^2^)] = 0.056	Hydrogen site location: inferred from neighbouring sites
*wR*(*F*^2^) = 0.136	H-atom parameters constrained
*S* = 1.08	*w* = 1/[σ^2^(*F*_o_^2^) + (0.0386*P*)^2^ + 0.5448*P*] where *P* = (*F*_o_^2^ + 2*F*_c_^2^)/3
2082 reflections	(Δ/σ)_max_ < 0.001
128 parameters	Δρ_max_ = 0.19 e Å^−^^3^
0 restraints	Δρ_min_ = −0.25 e Å^−^^3^

### Special details

**Table:**

Geometry. All e.s.d.'s (except the e.s.d. in the dihedral angle between two l.s. planes) are estimated using the full covariance matrix. The cell e.s.d.'s are taken into account individually in the estimation of e.s.d.'s in distances, angles and torsion angles; correlations between e.s.d.'s in cell parameters are only used when they are defined by crystal symmetry. An approximate (isotropic) treatment of cell e.s.d.'s is used for estimating e.s.d.'s involving l.s. planes.
Refinement. Refinement of *F*^2^ against ALL reflections. The weighted *R*-factor *wR* and goodness of fit *S* are based on *F*^2^, conventional *R*-factors *R* are based on *F*, with *F* set to zero for negative *F*^2^. The threshold expression of *F*^2^ > σ(*F*^2^) is used only for calculating *R*-factors(gt) *etc*. and is not relevant to the choice of reflections for refinement. *R*-factors based on *F*^2^ are statistically about twice as large as those based on *F*, and *R*- factors based on ALL data will be even larger.

### Fractional atomic coordinates and isotropic or equivalent isotropic displacement parameters (Å^2^)

**Table:**

	*x*	*y*	*z*	*U*_iso_*/*U*_eq_	
O2	0.5248 (4)	1.29754 (19)	0.09145 (5)	0.0460 (4)	
C10	0.8859 (5)	0.7924 (3)	0.12534 (7)	0.0351 (5)	
C1	0.6106 (5)	1.0740 (3)	0.14687 (7)	0.0389 (5)	
C9	0.7444 (5)	0.8979 (3)	0.15900 (7)	0.0347 (4)	
C2	0.6487 (5)	1.1357 (3)	0.09790 (7)	0.0369 (5)	
O3	1.0374 (5)	0.7687 (2)	0.04711 (5)	0.0549 (5)	
C5	1.0035 (6)	0.6270 (3)	0.13751 (8)	0.0423 (5)	
H5	1.0976	0.5566	0.1153	0.051*	
O1	0.4735 (5)	1.1666 (2)	0.17443 (6)	0.0636 (5)	
C3	0.7874 (5)	1.0345 (3)	0.06610 (7)	0.0393 (5)	
H3	0.8047	1.0774	0.0361	0.047*	
C6	0.9824 (6)	0.5657 (3)	0.18238 (8)	0.0483 (6)	
H6	1.0607	0.4542	0.1902	0.058*	
C4	0.9122 (5)	0.8589 (3)	0.07713 (7)	0.0384 (5)	
C7	0.8444 (6)	0.6705 (3)	0.21567 (8)	0.0482 (6)	
H7	0.8321	0.6295	0.2459	0.058*	
C8	0.7251 (6)	0.8354 (3)	0.20418 (7)	0.0426 (5)	
H8	0.6317	0.9049	0.2266	0.051*	
C11	0.5484 (6)	1.3721 (3)	0.04595 (8)	0.0508 (6)	
H11A	0.7849	1.3784	0.0388	0.076*	
H11B	0.4516	1.4874	0.0454	0.076*	
H11C	0.4243	1.3005	0.0233	0.076*	

### Atomic displacement parameters (Å^2^)

**Table:**

	*U*^11^	*U*^22^	*U*^33^	*U*^12^	*U*^13^	*U*^23^
O2	0.0583 (10)	0.0385 (8)	0.0422 (8)	0.0075 (7)	0.0122 (7)	−0.0003 (7)
C10	0.0342 (11)	0.0366 (11)	0.0347 (10)	−0.0011 (8)	0.0035 (8)	−0.0051 (8)
C1	0.0426 (12)	0.0375 (11)	0.0379 (11)	−0.0023 (9)	0.0124 (9)	−0.0080 (9)
C9	0.0341 (10)	0.0352 (10)	0.0354 (10)	−0.0059 (8)	0.0057 (8)	−0.0060 (8)
C2	0.0383 (11)	0.0331 (11)	0.0395 (11)	−0.0010 (8)	0.0053 (9)	−0.0030 (8)
O3	0.0747 (12)	0.0500 (10)	0.0418 (9)	0.0127 (9)	0.0185 (8)	−0.0096 (7)
C5	0.0440 (12)	0.0378 (11)	0.0453 (12)	0.0035 (9)	0.0037 (9)	−0.0076 (9)
O1	0.0953 (14)	0.0488 (10)	0.0502 (10)	0.0166 (9)	0.0325 (9)	−0.0034 (8)
C3	0.0444 (12)	0.0416 (11)	0.0324 (10)	0.0007 (9)	0.0070 (9)	−0.0023 (9)
C6	0.0534 (14)	0.0383 (12)	0.0528 (14)	0.0013 (10)	−0.0003 (11)	0.0024 (10)
C4	0.0372 (11)	0.0406 (12)	0.0379 (11)	−0.0007 (9)	0.0076 (9)	−0.0091 (9)
C7	0.0553 (14)	0.0495 (13)	0.0400 (12)	−0.0014 (11)	0.0037 (10)	0.0053 (10)
C8	0.0473 (13)	0.0440 (12)	0.0375 (11)	−0.0036 (10)	0.0102 (9)	−0.0049 (9)
C11	0.0607 (15)	0.0482 (13)	0.0442 (13)	0.0094 (11)	0.0090 (11)	0.0078 (10)

### Geometric parameters (Å, °)

**Table:**

O2—C2	1.340 (3)	C5—H5	0.9300
O2—C11	1.438 (3)	C3—C4	1.459 (3)
C10—C5	1.386 (3)	C3—H3	0.9300
C10—C9	1.402 (3)	C6—C7	1.385 (3)
C10—C4	1.489 (3)	C6—H6	0.9300
C1—O1	1.213 (2)	C7—C8	1.380 (3)
C1—C9	1.481 (3)	C7—H7	0.9300
C1—C2	1.504 (3)	C8—H8	0.9300
C9—C8	1.394 (3)	C11—H11A	0.9600
C2—C3	1.340 (3)	C11—H11B	0.9600
O3—C4	1.232 (2)	C11—H11C	0.9600
C5—C6	1.383 (3)		
			
C2—O2—C11	116.82 (16)	C5—C6—C7	119.9 (2)
C5—C10—C9	119.40 (19)	C5—C6—H6	120.0
C5—C10—C4	120.52 (18)	C7—C6—H6	120.0
C9—C10—C4	120.07 (19)	O3—C4—C3	120.4 (2)
O1—C1—C9	122.80 (19)	O3—C4—C10	121.1 (2)
O1—C1—C2	120.2 (2)	C3—C4—C10	118.50 (17)
C9—C1—C2	116.96 (16)	C8—C7—C6	120.3 (2)
C8—C9—C10	119.6 (2)	C8—C7—H7	119.8
C8—C9—C1	119.66 (18)	C6—C7—H7	119.8
C10—C9—C1	120.70 (18)	C7—C8—C9	120.1 (2)
O2—C2—C3	126.77 (19)	C7—C8—H8	119.9
O2—C2—C1	111.40 (16)	C9—C8—H8	119.9
C3—C2—C1	121.83 (19)	O2—C11—H11A	109.5
C6—C5—C10	120.6 (2)	O2—C11—H11B	109.5
C6—C5—H5	119.7	H11A—C11—H11B	109.5
C10—C5—H5	119.7	O2—C11—H11C	109.5
C2—C3—C4	121.89 (19)	H11A—C11—H11C	109.5
C2—C3—H3	119.1	H11B—C11—H11C	109.5
C4—C3—H3	119.1		
			
C5—C10—C9—C8	0.2 (3)	C4—C10—C5—C6	179.4 (2)
C4—C10—C9—C8	−179.15 (19)	O2—C2—C3—C4	−179.6 (2)
C5—C10—C9—C1	−178.86 (19)	C1—C2—C3—C4	−0.5 (3)
C4—C10—C9—C1	1.8 (3)	C10—C5—C6—C7	−0.4 (3)
O1—C1—C9—C8	−2.2 (3)	C2—C3—C4—O3	179.7 (2)
C2—C1—C9—C8	178.25 (19)	C2—C3—C4—C10	−0.5 (3)
O1—C1—C9—C10	176.9 (2)	C5—C10—C4—O3	0.2 (3)
C2—C1—C9—C10	−2.7 (3)	C9—C10—C4—O3	179.6 (2)
C11—O2—C2—C3	−1.2 (3)	C5—C10—C4—C3	−179.5 (2)
C11—O2—C2—C1	179.59 (18)	C9—C10—C4—C3	−0.1 (3)
O1—C1—C2—O2	1.7 (3)	C5—C6—C7—C8	0.6 (4)
C9—C1—C2—O2	−178.74 (17)	C6—C7—C8—C9	−0.3 (3)
O1—C1—C2—C3	−177.6 (2)	C10—C9—C8—C7	−0.1 (3)
C9—C1—C2—C3	2.0 (3)	C1—C9—C8—C7	179.0 (2)
C9—C10—C5—C6	0.0 (3)		
